# Supplementary material for: Mutations in PCYT2 disrupt etherlipid biosynthesis and cause a complex hereditary spastic paraplegia
Source: Brain. 2019 Oct 22;142(11):3382–97. doi: 10.1093/brain/awz291 (PMC6821184; doi:10.1093/brain/awz291)
Supplement: awz291_Supplementary_Data [file awz291_supplementary_data.zip › awz291-Suppl_data/Supplementary_Data4.zip!/Supplementary information 3 - fibroblast lipidomics.zip!/Fibroblast lipidomics/Controle vs Patient1/Statistical plots and data/PE species/Volcano plot_alternative.pdf]

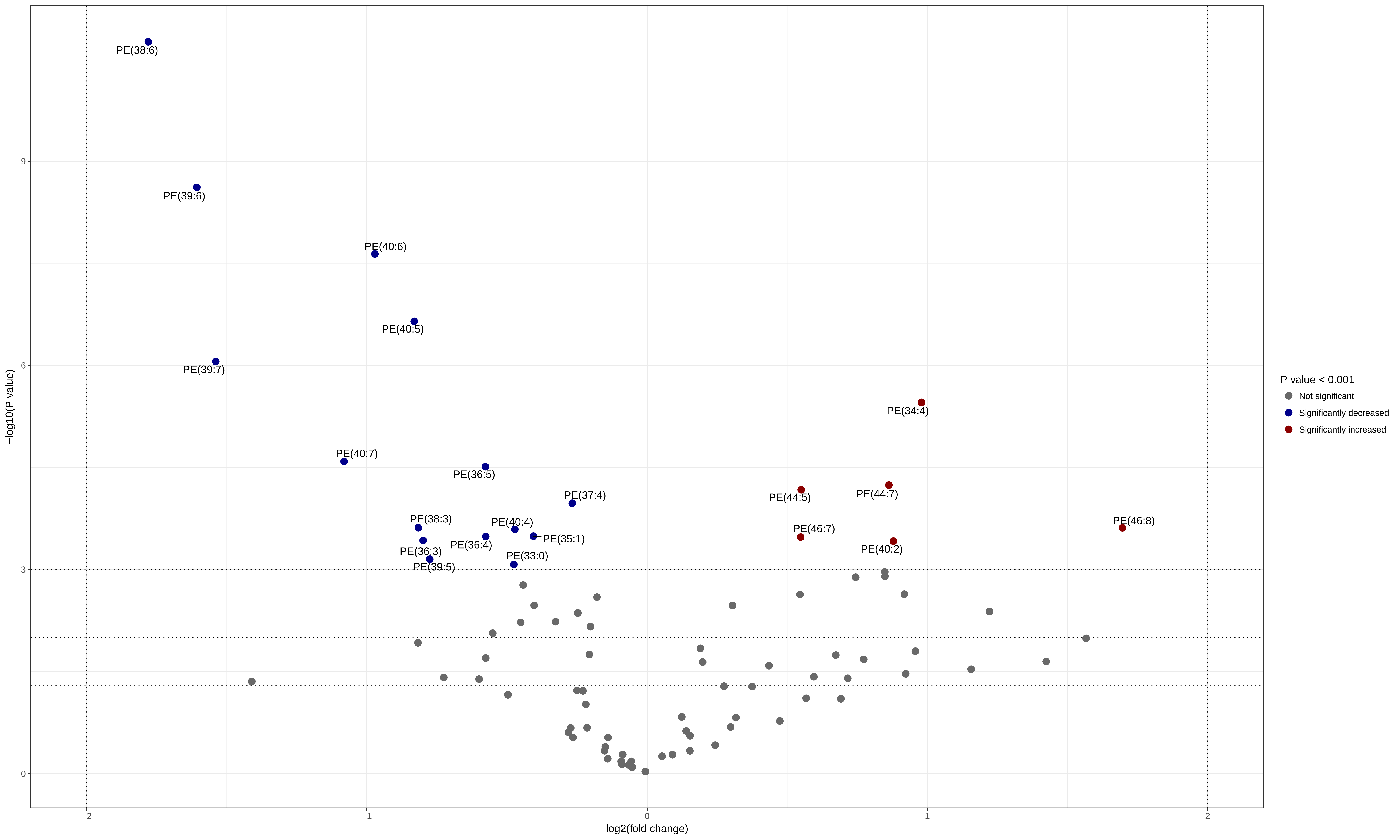

PE(38:6)
PE(39:6)
PE(40:6)
PE(40:5)
PE(39:7)
PE(40:7)
PE(36:5)
PE(37:4)
PE(38:3)
PE(40:4)
PE(35:1)
PE(36:4)
PE(36:3)
PE(33:0)
PE(39:5)
PE(34:4)
PE(44:5)
PE(44:7)
PE(46:8)
PE(46:7)
PE(40:2)
9
6
3
0
−log10(P value)
−2
−1
0
1
2
log2(fold change)
P value < 0.001
Not significant
Significantly decreased
Significantly increased
